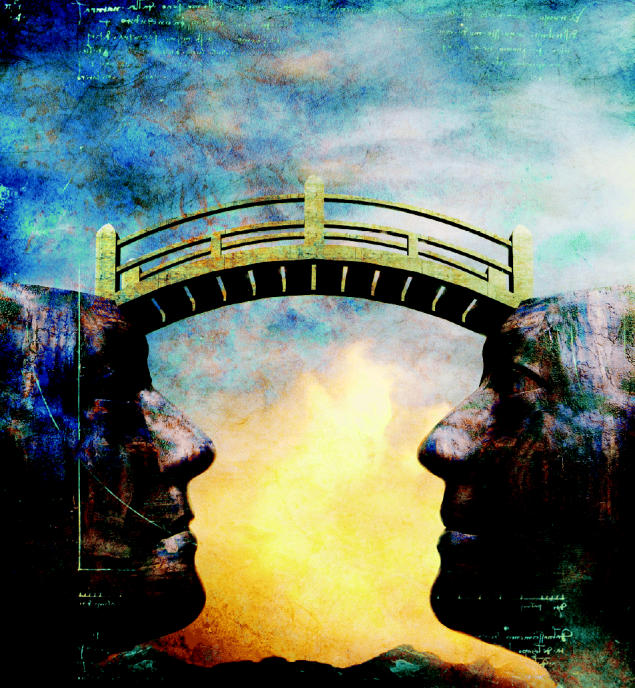# New Program to Identify Outstanding New Investigators

**Published:** 2005-10

The National Institute of Environmental Health Sciences as launched an innovative new program of R01 research grants specifically designed for new investigators to support their research and to enhance their career progression: the Outstanding New Environmental Scientist (ONES) Award. Through this program, the NIEHS hopes to identify talented scientists who are in the early, formative stages of their career, who have not yet been awarded their first R01 grant support, and who intend to make a long-term career commitment to research related to the mission of the NIEHS.

These awards will offer several unique features to make them attractive to new investigators:

- Funds earmarked for program ($3.6 million)
- Review by a Special Emphasis Panel with scientific expertise targeted to the mission of the NIEHS and to the applicant pool
- A unique budget structure that will allow applicants to request up to $250,000 base funds in all 5 years, plus an additional $150,000 per year in years 1–2 and $25,000 in years 3–5 for equipment or other resource development and/or career enhancement activities.

The ONES award is designed to be highly competitive. In addition to the unique features outlined above, only a limited number—six in fiscal year 2006—will be awarded. In addition, to further encourage universities to identify their best new investigators as potential applicants, only one application per school or college within an institution will be accepted.

The mission of the NIEHS is distinguished from that of other Institutes by its focus on research programs seeking to link the effects of environmental exposures to the cause, mechanisms, moderation, or prevention of a human disease, disorder, or relevant pathophysiologic process. Proposals will have to address research topics that fall within this mission.

Letters of intent are due 21 November 2005, and applications are due on 21 December 2005.

For the full text of the announcement, please refer to the NIH Guide for Grants and Contracts at http://grants.nih.gov/grants/guide/rfa-files/RFA-ES-05-005.html.

## Contact

**Dr. Carol Shreffler** | 919-541-1445, shreffl1@niehs.nih.gov

**Dr. Pat Mastin** | 919-541-3289, mastin@niehs.nih.gov

## Figures and Tables

**Figure:**